# Blocking CD248 molecules in perivascular stromal cells of patients with systemic sclerosis strongly inhibits their differentiation toward myofibroblasts and proliferation: a new potential target for antifibrotic therapy

**DOI:** 10.1186/s13075-018-1719-4

**Published:** 2018-10-03

**Authors:** Paola Di Benedetto, Vasiliki Liakouli, Piero Ruscitti, Onorina Berardicurti, Francesco Carubbi, Noemi Panzera, Salvatore Di Bartolomeo, Giuliana Guggino, Francesco Ciccia, Giovanni Triolo, Paola Cipriani, Roberto Giacomelli

**Affiliations:** 1 Department of Biotechnological and Applied Clinical Sciences, Rheumatology Unit, School of Medicine, University of L’Aquila, Delta 6 Building, Via dell’Ospedale, 67100 L’Aquila, Italy; 2 Department of Internal Medicine, Division of Rheumatology, University of Palermo, Piazza delle Cliniche 2, 90127 Palermo, Italy

**Keywords:** Systemic sclerosis, CD248, Fibrosis

## Abstract

**Background:**

Fibrosis may be considered the hallmark of systemic sclerosis (SSc), the end stage triggered by different pathological events. Transforming growth factor-β (TGF-β) and platelet-derived growth factor BB (PDGF-BB) are profibrotic molecules modulating myofibroblast differentiation and proliferation, respectively. There is evidence linking CD248 with these two molecules, both highly expressed in patients with SSc, and suggesting that CD248 may be a therapeutic target for several diseases. The aim of this work was to evaluate the expression of CD248 in SSc skin and its ability to modulate SSc fibrotic process.

**Methods:**

After ethical approval was obtained, skin biopsies were collected from 20 patients with SSc and 10 healthy control subjects (HC). CD248 expression was investigated in the skin, as well as in bone marrow mesenchymal stem cells (MSCs) treated with TGF-β or PDGF-BB, by immunofluorescence, qRT-PCR, and Western blotting. Finally, in SSc-MSCs, the CD248 gene was silenced by siRNA.

**Results:**

Increased expression of CD248 was found in endothelial cells and perivascular stromal cells of SSc skin. In SSc-MSCs, the levels of CD248 and α-smooth muscle actin expression were significantly higher than in HC-MSCs. In both SSc- and HC-MSCs, PDGF-BB induced increased expression of *Ki-67* when compared with untreated cells but was unable to modulate CD248 levels. After CD248 silencing, both TGF-β and PDGF-BB signaling were inhibited in SSc-MSCs.

**Conclusions:**

CD248 overexpression may play an important role in the fibrotic process by modulating the molecular target, leading to perivascular cells differentiation toward myofibroblasts and interfering with its expression, and thus might open a new therapeutic strategy to inhibit myofibroblast generation during SSc.

## Background

CD248 (also known as *endosialin* or *tumor endothelial marker 1*) is a transmembrane receptor whose known ligands are fibronectin and type I/IV collagen. It is widely expressed on mesenchymal cells during embryonic life and is required for proliferation and migration of pericytes and fibroblasts [1]. Although CD248 expression is dramatically reduced during adult life, it may be upregulated during specific conditions such as malignancy, inflammation, and fibrosis [2–4]. It is well known that CD248 is expressed on the surface of cells of mesenchymal origin, including tumor-associated pericytes and activated fibroblasts, which are thought to play a key role in the development of tumor neovascular networks and stromal interaction [1]. The interruption of endosialin function, with antibody blockade or genetic knockouts, negatively affects tumor growth and angiogenesis in numerous cancer types [5–7]. Furthermore, in the experimental model of kidney fibrosis after unilateral ureteral obstruction (UUO), CD248^−/−^ mice display downregulation of myofibroblast proliferation, thus decreasing the kidney fibrosis [8]. These biologic effects, in cancer and in reparative response, may be related to the ability of CD248 to modulate many signaling pathways involved in both cancer development and tissue repair, including platelet-derived growth factor BB (PDGF-BB), transforming growth factor-β (TGF-β), and Notch receptor protein [9]. Under normal conditions, pericytes that expressed high levels of CD248 were able to proliferate, responding to PDGF-BB stimulation [9], and higher expression of CD248 is required for imparting fibroblast sensitivity to the effects of TGF-β [10].

Owing to its multifunctional activities modulating innate immunity, cell proliferation, and vascular homeostasis [9, 11], CD248 may be considered a potential therapeutic target for several diseases [12], and currently, the results of a first-in-human, open-label, phase I study recruiting patients with extracranial solid tumors who failed standard chemotherapy and were treated with a biologic therapy targeting CD248 have been published, confirming the therapy’s safety and a positive impact on different cancers [13].

Systemic sclerosis (SSc) is a connective tissue disease of unknown etiology with multiorgan involvement and heterogeneous clinical manifestations. The hallmark of early SSc is endothelial involvement, whereas later stages are characterized by an excessive accumulation of extracellular matrix (ECM), resulting in extended fibrosis in skin and internal organs [14, 15]. In the last few years, it has been clarified that endothelial cells (ECs) and pericytes, after injury, may differentiate toward myofibroblasts, which are committed to producing increased amounts of collagen [16–18], and this process has been proposed as a key pathogenic mechanism in SSc.

Several polypeptide mediators are involved in fibrosis during SSc, such as TGF-β and PDGF-BB. The latter is a potent pro-proliferative signal for mesenchyme-derived cells, including myofibroblasts [19, 20], whereas TGF-β primarily promotes myofibroblast activation, α-smooth muscle actin (α-SMA) expression, and collagen deposition [16, 21–26]. Interestingly, CD248 modulates both these pathways because of CD248 is required for imparting fibroblast sensitivity to the effects of TGF-β [9] and is crucial for optimal migratory response of activated fibroblasts to PDGF-BB [19].

The goal of this work is to investigate the expression of CD248 in skin perivascular stromal cells from patients with SSc and its ability in mediating pericyte differentiation toward myofibroblasts. Although the role of CD248 in the pathogenesis of SSc has not yet been established, its potential role in controlling vessel regression and fibrosis makes this molecule a potential therapeutic target in a clinical setting, different from cancer, and in which an effective therapeutic approach to prevent fibrosis is still an important unmet need.

## Methods

### Patients, control subjects, and skin biopsies

Full-thickness biopsy samples measuring 2 × 0.5 cm isolated from excisional biopsy were obtained from clinically involved skin of one-third of the distal forearm of patients with diffuse SSc according to the classification of LeRoy and colleagues [27]. All patients fulfilled the 2013 classification criteria for SSc [28]. Skin with a modified Rodnan skin score [29] ≥ 1 was considered to be clinically involved.

To be sure that 50% of our patients were in a very early phase of SSc, considering that the term *early* currently refers to an undifferentiated connective tissue disease at higher risk of developing into scleroderma, as suggested by the pivotal study of Koening et al. [30], more than to a time frame from the beginning of the disease, we further divided our patients into two subsets: patients fulfilling the classification criteria in less than 1 year from the onset of Raynaud’s phenomenon (early-onset subset [EOS], *n* = 10) and all the others (long-standing subset [LSS], *n* = 10). Skin samples from the same region of ten age- and sex-matched healthy control subjects (HC) who underwent a surgical treatment for trauma were used for comparison. All patients with SSc underwent a 20-day washout from any immunosuppressive treatment and 1 month from intravenous prostanoids before skin biopsy was performed. During this period, only proton pump inhibitors and clebopride were allowed. Patients who could not undergo therapeutic washout owing to severe organ complications were not enrolled in the study. Biopsies were taken after informed consent was obtained, and the study was approved by our local ethics committee (ASL Avezzano Sulmona L’Aquila, protocol number 015408/17). Demographic and clinical characteristics of the patients are shown in Table 1. Each biopsy sample was divided into specimens for immunofluorescence (IF) and qRT-PCR. For IF, the specimens were fixed in 10% buffered formalin, dehydrated in graded alcohol series, and embedded in paraffin. Specimens used for qRT-PCR analyses were immediately immersed in liquid nitrogen and stored at − 80 °C until use.

**Table 1:** Clinical and demographic features of the 20 patients with diffuse systemic sclerosis

Sex/age (yr)	Disease duration at skin biopsy (yr from RP)	mRSS/score at skin biopsy	Autoantibodies	ILD	PAH	SRC	RP	DU
95% F	50% EOS 50% LSS		100% ANA/Scl70	25% ILD	15% PAH;	0% SCR	100% RP	30% DU
F/45	< 1	10/2	ANA/Scl-70	No	No	No	Yes	No
F/22	< 1	13/1	ANA/Scl-70	No	No	No	Yes	Yes
F/31	< 1	08/2	ANA/Scl-70	No	No	No	Yes	No
F/38	< 1	09/2	ANA/Scl-70	No	Yes	No	Yes	Yes
M/20	< 1	11/1	ANA/Scl-70	No	No	No	Yes	No
F/40	< 1	10/2	ANA/Scl-70	No	No	No	Yes	No
F/31	< 1	10/1	ANA/Scl-70	No	No	No	Yes	No
F/21	< 1	09/1	ANA/Scl-70	No	No	No	Yes	No
F/31	< 1	14/1	ANA/Scl-70	No	No	No	Yes	No
F/42	< 1	16/2	ANA/Scl-70	Yes	No	No	Yes	No
F/45	4	17/2	ANA/Scl-70	No	No	No	Yes	No
F/21	5	15/1	ANA/Scl-70	No	No	No	Yes	No
F/30	6	18/2	ANA/Scl-70	No	Yes	No	Yes	Yes
F/33	4	13/2	ANA/Scl-70	Yes	No	No	Yes	No
F/34	4	12/1	ANA/Scl-70	No	No	No	Yes	Yes
F/40	4	10/2	ANA/Scl-70	Yes	Yes	No	Yes	No
M/26	6	10/2	ANA/Scl-70	Yes	No	No	Yes	Yes
F/21	4	11/1	ANA/Scl-70	No	No	No	Yes	No
F/30	3	12/2	ANA/Scl-70	Yes	No	No	Yes	Yes
F/33	4	12/1	ANA/Scl-70	No	No	No	Yes	No

*Abbreviations: EOS* Early-onset subset, *LSS* Long-standing subset, *RP* Raynaud’s phenomenon, *mRSS* Modified Rodnan skin score (maximum possible score 51), *ILD* Interstitial lung disease, *ANA* Antinuclear antibodies, *Scl-70* Antitopoisomerase, *PAH* Pulmonary arterial hypertension, *SRC* Scleroderma renal crisis, *DU* Digital ulcers

The internal organ involvement is referred to the time of biopsies

### Immunofluorescence

The IF analysis was performed on paraffin sections (thickness 3 μm) using a conjugated anti-CD248 antibody (Novus Biologicals, Littleton, CO, USA). Antigen retrieval was carried out using Dako Target Retrieval solution (Agilent Technologies, Santa Clara, CA, USA). Vasculature pericytes were highlighted using a Cy3-conjugated anti-α-SMA antibody (Sigma-Aldrich, St. Louis, MO, USA) and EC using unconjugated anti–von Willebrand factor (vWF) antibody (Dako, Glostrup, Denmark). The immunoreaction was revealed using secondary antibody (Alexa Fluor; Life Technologies, Carlsbad, CA, USA). Cell nuclei were visualized using 4′,6-diamidino-2-phenylindole. Fluorescence was analyzed using a BX53 fluorescence microscope (Olympus, Center Valley, PA, USA). The intensity of fluorescence was measured using ImageJ software (National Institutes of Health, Bethesda, MD, USA).

### Isolation, culture, and immunophenotyping of mesenchymal stem cells

After approval was provided by the local ethics committee (ASL Avezzano Sulmona L’Aquila) and written informed consent was obtained from patients, bone marrow was obtained by aspiration from the posterior superior iliac crest from the patients enrolled in the study. Samples of mesenchymal stem cells (MSC) from bone marrow donors were used as a control. MSC were obtained and expanded from both the subsets of five EOS and five LSS patients, as previously described [25]. Third-passage MSC were analyzed for the surface expression of MSC antigens (CD45, CD73, CD90, CD34, CD79a, PDGF receptor-β) and pericyte markers (α-SMA, SM22α, NG2, desmin, RGS5) by flow cytometry (FACScan; BD Biosciences, San Jose, CA, USA) as previously described [25] (data not shown).

### MSC response to PDGF-BB and TGF-β

To establish the optimal concentration of PDGF-BB and TGF-β molecules, in our system, a dose-response curve was created, using P3 cells of one HC and one patient and evaluating the Ki-67 and α-SMA messenger RNA (mRNA) expression. Each experiment was performed in triplicate (data not shown). Both SSc-MSC and HC-MSC were cultured for 7 days in 1% FBS medium supplemented with selected doses of 10 ng/ml PDGF-BB (R&D Systems, Minneapolis, MN, USA) and 10 ng/ml TGF-β (R&D Systems). We used the TGF-β1 isoform. Media were changed every 2 days.

### Small interfering RNA assay

To silence CD248 expression, SSc-MSC were transfected with Silencer Select CD248 small interfering RNA (siRNA; Life Technologies) [31] or with Silencer Select nontargeting scrambled (scr) siRNA (Life Technologies) using Lipofectamine™ 2000 reagent (Life Technologies). The CD248 gene was identified to be transcribed from a 2557-bp single-exon gene [32, 33].

Transfection was performed according to the manufacturer’s instructions. Briefly, MSC were plated at 1 × 10^4^ cells/cm^2^ 24 h prior to transfection. Cultures were incubated for 24 h with 30 pmol of siRNA in 2 ml of Opti-MEM (Life Technologies). After incubation, plates were washed, and cells were allowed to recover in growth conditions (1% FBS) supplemented with TGF-β (10 ng/ml) and PDGF-BB (10 ng/ml).

### Western blot analysis

MSC, derived from two EOS and two LSS patients, were pelleted and lysed in lysis buffer (radioimmunoprecipitation assay buffer; Cell Signaling Technology, Danvers, MA, USA) for 30 min and cleared by centrifugation. The protein concentration was calculated by using a bicinchoninic acid protein assay kit (EuroClone, Pero, Italy). Proteins (40 μg) were separated by SDS-PAGE and transferred to nitrocellulose membranes. After blocking in 10% nonfat milk in Tris-buffered saline/1% Tween 20 and incubated with the primary antibodies α-SMA (Santa Cruz Biotechnology, Dallas, TX, USA) and CD248 (Novus Biologicals), horseradish peroxidase-conjugated secondary antibodies (Cell Signaling Technology) were appropriately used. The detection was performed using long-lasting chemiluminescent substrate (EuroClone). All the signals were quantified by normalizing to the β-actin signal (Santa Cruz Biotechnology). Immunoreactive bands were acquired using a ChemiDoc imaging system with Image Lab software (Bio-Rad Laboratories, Hercules, CA, USA) and quantified with densitometry using ImageJ software.

### qRT-PCR analysis

Total RNA was extracted from MSC and whole biopsy using NucleoSpin RNA (Macherey-Nagel, Düren, Germany) and reverse-transcribed into complementary DNA (cDNA) with the High Capacity cDNA Reverse Transcription Kit (Applied Biosystems, Foster City, CA, USA). The qRT-PCR was run in triplicate. *Ki-67* and *GAPDH* gene expression were assessed using commercial TaqMan gene expression assay (Hs01032443_m1; Hs02758991_g1, respectively). * α-SMA*, *CD248*, and *β-actin *expression was performed by using SYBR Green kits (Applied Biosystems). Primers were designed on the basis of reported sequences in the National Center for Biotechnology Information PrimerBank [β-actin: 5′-CCTGGCACCCAGCACAAT-3′ (forward) and 5′-AGTACTCCGTGTGGATCGGC-3′ (reverse); α-SMA: 5′-CGGTGCTGTCTCTCTATGCC-3′ (forward) and 5′-CGCTCAGTCAGGATCTTCA-3′ (reverse); CD248: 5′-AGTGTTATTGTAGCGAGGGACA-3′ (forward) and 5′-CCTCTGGGAAGCTCGGTCTA-3′ (reverse)]. Results were analyzed after 45 cycles of amplification using the ABI 7500 Fast Real Time PCR System (Applied Biosystems).

### Statistical analysis

Prism 5.0 software (GraphPad Software, La Jolla, CA, USA) was used for statistical analyses. Results are expressed as median (range). Owing to the nonparametric distribution of our data, the Mann-Whitney *U* test was used as appropriate for analyses. Statistical significance was expressed by a *p* value < 0.05.

## Results

### CD248 expression in skin SSc

**Fig. 1:**
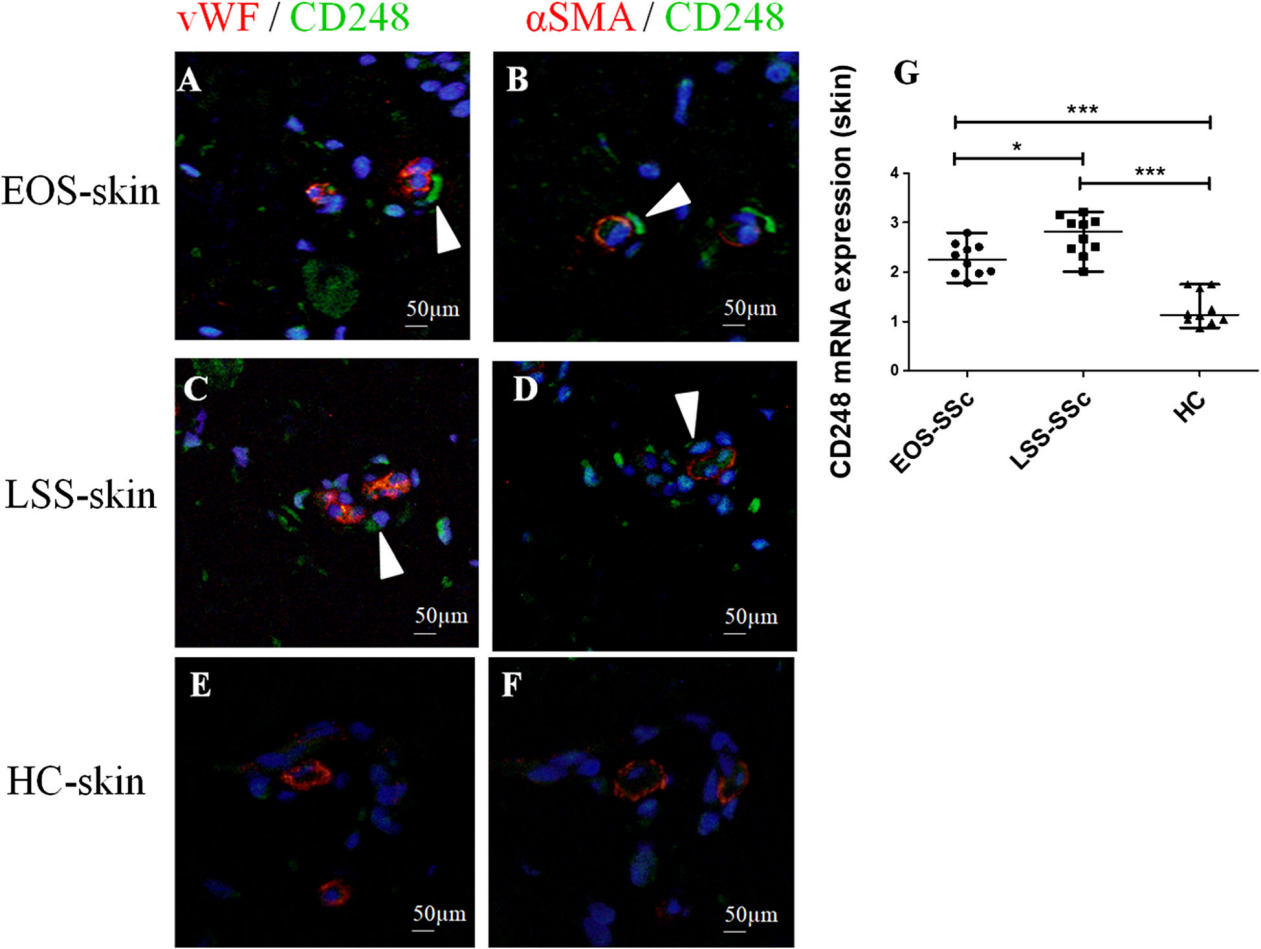
CD248 expression in skin of patients with systemic sclerosis (SSc). **a**, **b** Immunofluorescence staining of ten early-onset subset (EOS) SSc skin samples. **a** CD248 (*green*) and von Willebrand factor (vWF) (*red*) staining. **b** Consecutive section stained with CD248 (*green*), α-smooth muscle actin (α-SMA) (*red*). CD248 is expressed in endothelial cells (EC) and perivascular cells of EOS SSc skin vessels. The *arrowheads* show CD248^+^ cells localized close to microvessels. **c** and **d** Immunofluorescence staining of ten long-standing subset (LSS) SSc skin samples. **c** CD248 (*green*) and vWF (*red*) staining. **d** Consecutive section stained with CD248 (*green*) and α-SMA (*red*). CD248 is expressed in EC and perivascular cells of LSS SSc skin vessels. The *arrowheads* show CD248^+^ cells localized close to microvessels. **e** and **f** Immunofluorescence staining of ten healthy control subject (HC) skin samples. Microphotographs show (**e**) CD248 (*green*) and vWF (*red*) staining and (**f**) consecutive section stained with CD248 (*green*) and α-SMA (*red*). Weak expression of CD248 may be observed in EC and pericytes of HC skin vessels. Negative control samples were obtained by omitting the primary antibody. Original magnification × 20. **g** qRT-PCR of CD248 messenger RNA (mRNA) levels in ten EOS-SSc skin, ten LSS-SSc skin, and ten HC-skin samples. In SSc-skin, CD248 mRNA expression levels are always significantly higher than in HC mesenchymal stem cells. CD248 mRNA expression is significantly higher in LSS-SSc skin than in EOS-SSc skin. * *p* = 0.01, *** *p* < 0.0001

Our results show that CD248 is overexpressed in SSc skin, and specifically in both EC and perivascular cells, when compared with HC skin, as observed in Fig. 1. Consistent with these findings, in whole SSc skin biopsies, CD248 mRNA expression was significantly increased when compared with HC skin, as assessed by qRT-PCR. Furthermore, in LSS skin, the CD248 mRNA expression was significantly increased when compared with EOS skin (Fig. 1g).

**Fig. 2:**
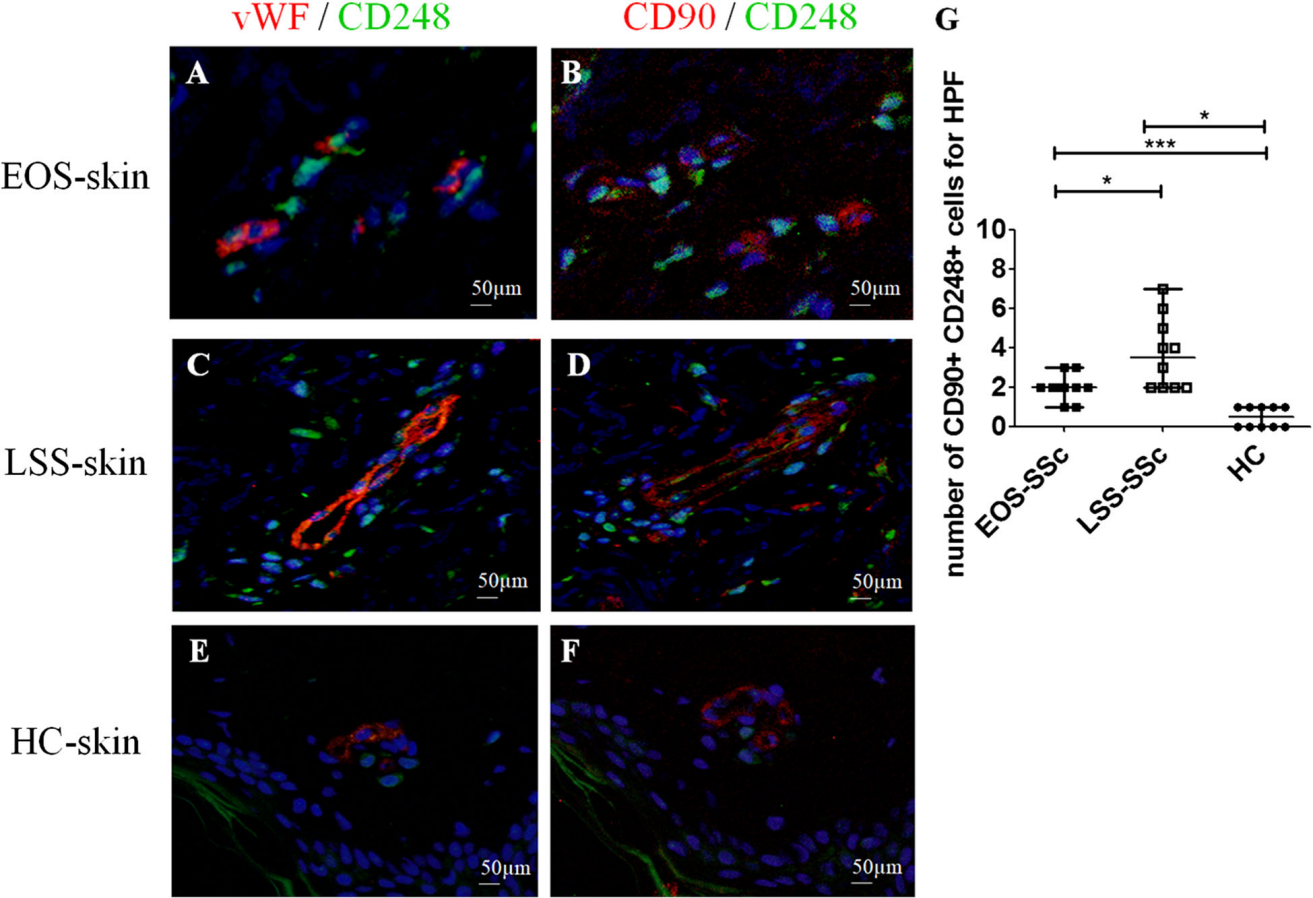
CD248^+^/CD90^+^ mesenchymal stem cells (MSCs) surrounding the vessels in systemic sclerosis (SSc) skin. **a**, **b** Immunofluorescence staining of ten early-onset subset (EOS) SSc skin samples. Microphotographs show (**a**) CD248 (*green*) and von Willebrand factor (vWF) (*red*) staining and (**b**) consecutive section stained with CD248 (*green*) and CD90 (*red*). **c** and **d** Immunofluorescence staining of ten long-standing subset (LSS) SSc skin samples. Microphotographs show (**c**) CD248 (*green*) and vWF (*red*) staining and (**d**) consecutive section stained with CD248 (*green*) and CD90 (*red*). **e** and **f** Immunofluorescence staining of ten healthy control subject (HC) skin samples. Microphotographs show (**e**) CD248 (*green*) and vWF (*red*) staining and (**f**) consecutive section stained with CD248 (*green*) and CD90 (*red*). Negative controls were obtained by omitting the primary antibody. Original magnification × 20. **g** Median number of CD90^+^/CD248^+^ cells. The number of CD90^+^/CD248^+^ cells is significantly higher in LSS-SSc skin than in EOS-SSc skin. Any dot plot is representative of the median cell count per 5 high-power fields (HPF) (× 40) for each patient. * *p* = 0.02, *** *p* = 0.0001

Interestingly, we observed that CD248 expression was not limited only to the cells of blood vessels; also other cells, proximal to microvessels, showed an increased expression of this marker (Fig. 1a–d, arrowheads). To better understand our findings, we performed different staining to identify the possible lineage of these cells, and as shown in Fig. 2, these cells surrounding the vascular trees coexpressed the CD90 marker, which is highly expressed in undifferentiated MSC [34]. Of interest, these CD90^+^/CD248^+^ cells were significantly increased in SSc skin when compared with HC skin (Fig. 2). Finally, the number of these CD90^+^/CD248^+^ cells was significantly higher in LSS SSc skin (Fig. 2c and d) than in EOS SSc skin (Fig. 2a and b).

### TGF-β and PDGF-BB effects on CD248 expression in MSC

**Fig. 3:**
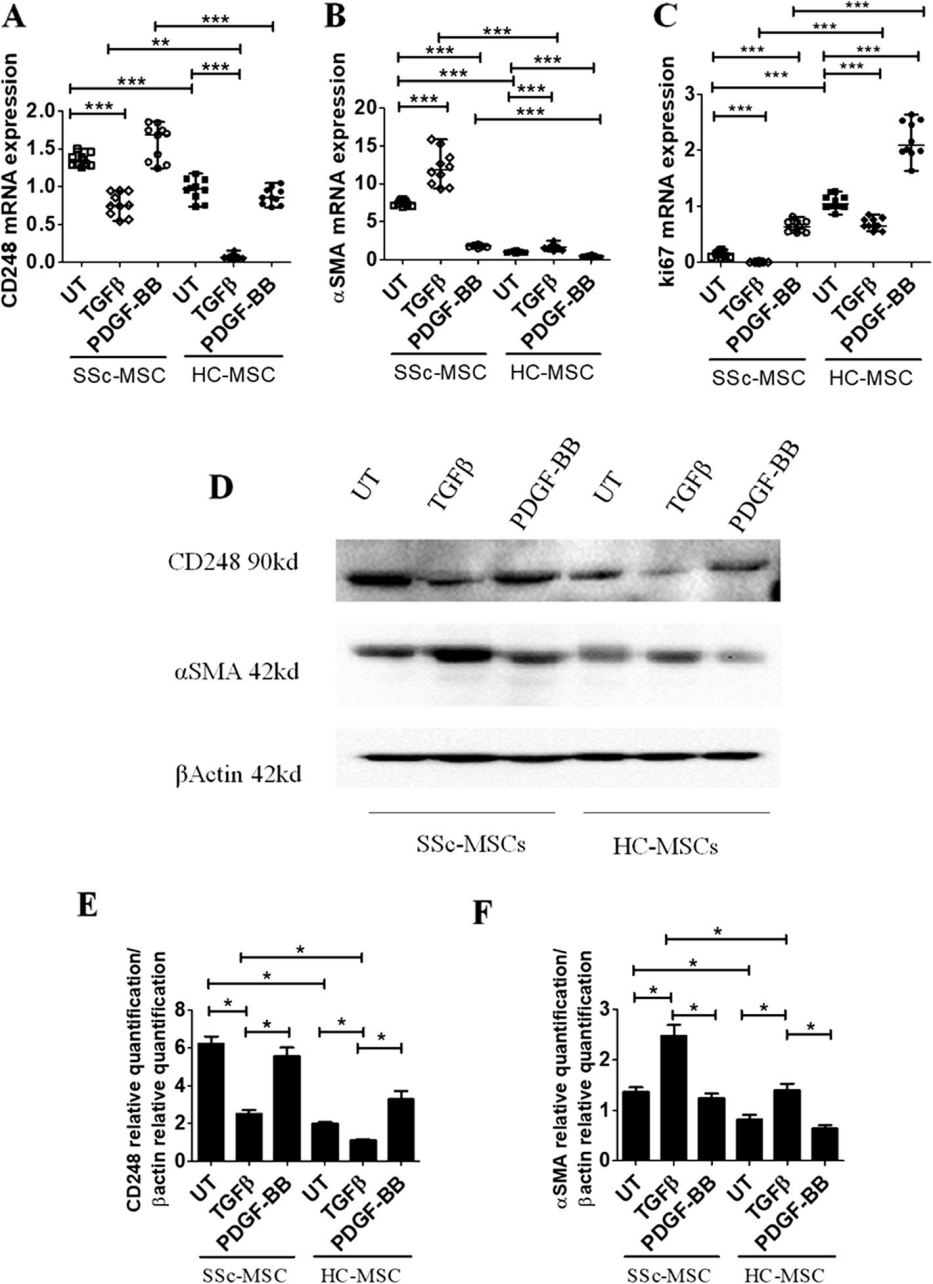
Transforming growth factor (TGF)-β and platelet-derived growth factor (PDGF)-BB effects on CD248, α-smooth muscle actin (α-SMA), and Ki-67 expression in systemic sclerosis (SSc) mesenchymal stem cells (MSCs). **a** qRT-PCR of CD248 messenger RNA (mRNA) levels in ten SSc-MSC (five early-onset subset [EOS] and five long-standing subset [LSS]) and ten healthy control subject (HC) MSC samples. In SSc-MSCs, CD248 mRNA expression levels are always significantly higher than in HC-MSCs. **b** qRT-PCR of α-SMA mRNA levels in ten SSc-MSCs (five EOS and five LSS) and ten HC-MSCs. In SSc-MSCs, the α-SMA mRNA levels are always significantly higher than in HC-MSCs. In both SSc- and HC-MSCs, TGF-β treatment induces a significant increase of α-SMA mRNA expression compared with untreated (UT) cells. On the contrary, PDGF-BB treatment induces a significant decrease of α-SMA compared with UT cells in both HC- and SSc-MSCs. **c** qRT-PCR of Ki-67 mRNA levels in ten SSc-MSCs (five EOS and five LSS) and ten HC-MSCs. In both SSc- and HC-MSCs, TGF-β treatment induces a significant decrease of Ki-67; on the contrary, PDGF-BB induces a significant increase of Ki-67 when compared with UT cells in both SSc- and HC-MSCs. The TGF-β isoform used is TGF-β1. Any single dot in the figure represents the median of triplicate experiments for each patient ** *p* = 0.0002, *** *p* = 0.0001. **d** Western blot analyses performed in four SSc-MSCs (two EOS and two LSS) and four HC SSc-MSCs confirmed the results observed by qRT-PCR analyses. Pictures are representative of all experiments. **e** and **f** Densitometric analysis of (**e**) CD248 protein bands and (**f**) α-SMA protein bands. The values were expressed as protein relative quantification/β-actin relative quantification. * *p* = 0.02

We investigated the functional role of CD248 in vitro in the perivascular differentiation toward myofibroblasts, by using MSC, a validated surrogate of perivascular cells [26]. Figure 3a shows that in SSc-MSC, CD248 mRNA expression levels were significantly higher than in HC-MSC [CD248 mRNA levels in untreated [UT] SSc-MSC 1.32 (1.25–1.50) vs UT HC-MSC 0.96 (0.73–1.17); *p* < 0.0001]. Furthermore, in SSc-MSC, TGF-β induced a significant decrease of CD248 mRNA expression levels when compared with UT SSc-MSC [CD248 mRNA levels in TGF-β SSc-MSC 0.74 (0.54–0.94) vs UT SSc-MSC 1.32 (1.25–1.50); *p* < 0.0001]. In HC-MSC, the TGF-β treatment induced a significant reduction of CD248 mRNA expression levels when compared with UT cells [CD248 mRNA levels in TGF-β HC-MSC 0.07 (0.03–0.15) vs UT HC-MSC 0.96 (0.73–1.17); *p* < 0.0001], although in SSc-MSC, the levels of CD248 expression were always significantly higher than in HC-MSC. On the contrary, no CD248 mRNA modulation was observed by using PDGF-BB, in both SSc- and HC-MSC [CD248 mRNA levels in PDGF-BB SSc-MSC 1.69 (1.24–1.85) vs UT SSc-MSC 1.32 (1.25–1.50); *p* = ns; CD248 mRNA levels in PDGF-BB HC-MSC 0.85 (0.72–1.0) vs UT HC-MSC 0.96 (0.73–1.17); *p* = n.s.].

### TGF-β induces an increase of α-SMA in MSC

TGF-β treatment induced a significant increase of α-SMA mRNA expression in both SSc- and HC-MSC when compared with UT cells [α-SMA mRNA levels in TGF-β SSc-MSC 11.90 (9.36–15.87) vs UT SSc-MSC 7.53 (6.94–7.94); *p* < 0.0001; α-SMA mRNA levels in TGF-β HC-MSC 1.66 (1.14–2.48) vs UT HC-MSC 1.05 (0.84–1.18); *p* < 0.0001]. On the contrary, PDGF-BB treatment induced a significant decrease of α-SMA when compared with UT cells, in both HC- and SSc-MSC [α-SMA mRNA levels in PDGF-BB SSc-MSC 1.84 (1.52–2.01) vs UT SSc-MSC 7.53 (6.94–7.94); *p* < 0.0001; α-SMA mRNA levels in PDGF-BB HC-MSC 0.54 (0.24–0.70) vs UT HC-MSC 1.05 (0.84–1.18); *p* < 0.0001] (Fig. 3b). Western blot analysis confirmed the results of gene expression (Fig. 3d).

### TGF-β and PDGF-BB effects on cell proliferation in MSC

To assess the proliferative ability of our cells, we performed qRT-PCR for Ki-67 gene expression, a molecule considered to be associated with active proliferation. We observed that TGF-β treatment induced a significant decrease of Ki-67 in both SSc- and HC-MSC; on the contrary, PDGF-BB induced a significant increase of Ki-67 when compared with UT cells in both SSc- and HC-MSC [Ki-67 mRNA levels in TGF-β SSc-MSC 0.0066 (0.0013–0.016) vs UT SSc-MSC 0.16 (0.08–0.23); *p* < 0.0001; Ki-67 mRNA levels in TGF-β HC-MSC 0.64 (0.54–0.84) vs UT HC-MSC 1.04 (0.85–1.26); *p* < 0.0001; Ki-67 mRNA levels in PDGF-BB SSc-MSC 0.63 (0.52–0.81) vs UT SSc-MSC 0.16 (0.08–0.23); *p* < 0.0001; Ki-67 mRNA levels in PDGF-BB HC-MSC 2.08 (1.63–2.63) vs UT HC-MSC 1.04 (0.85–1.26); *p* < 0.0001] (Fig. 3c).

### CD248 silencing interferes with PDGF-BB and TGF-β signaling in SSc-MSC

**Fig. 4:**
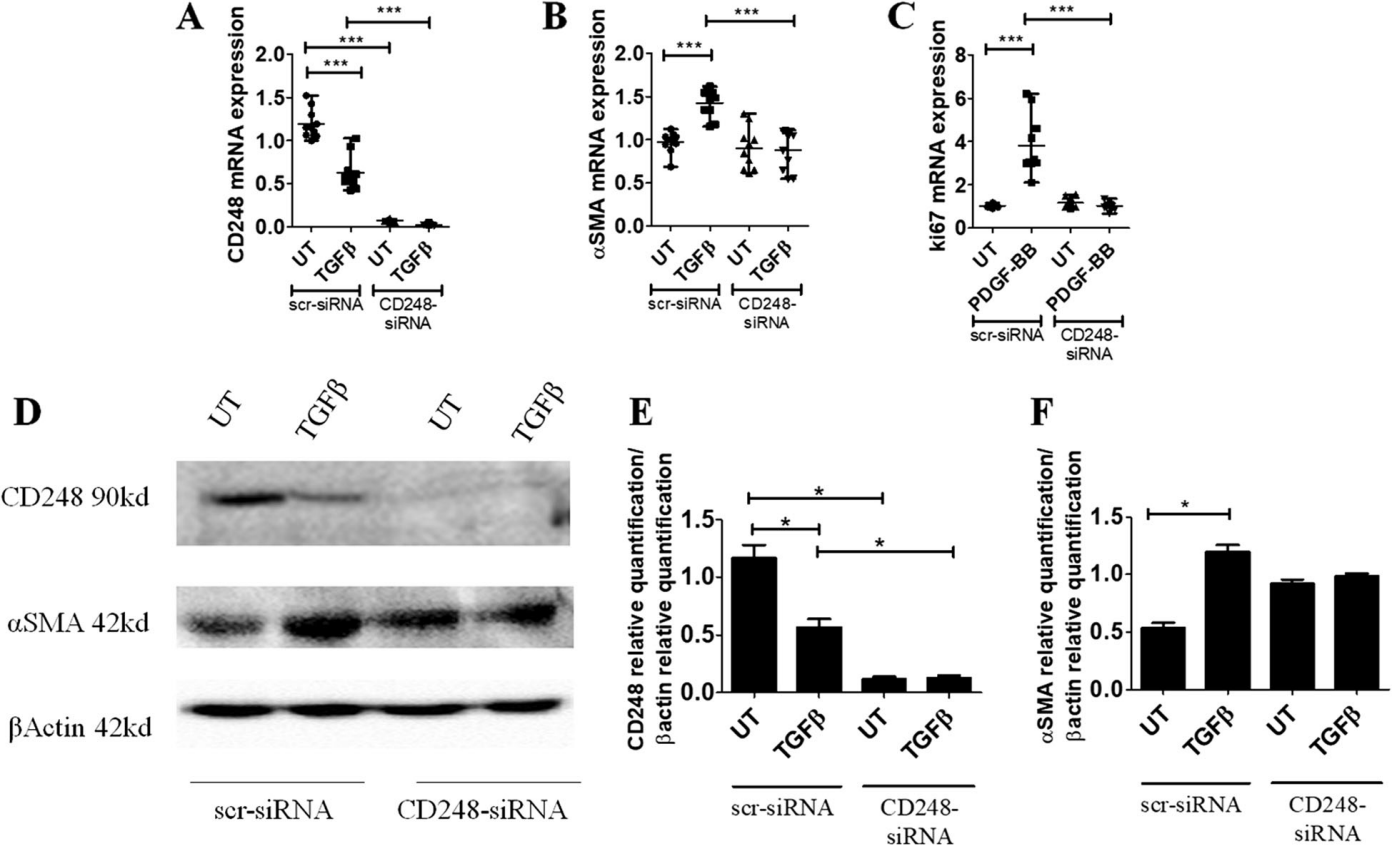
Systemic sclerosis (SSc) mesenchymal stem cells (MSCs) silenced by small interfering RNA (siRNA) CD248. **a** qRT-PCR of CD248 in ten SSc-MSCs (five early-onset subset [EOS] and five long-standing subset [LSS]) transfected with specific CD248-siRNA or scrambled (scr)-siRNA. Cells transfected with CD248-siRNA show a decreased expression of the CD248 gene compared with cells transfected with scr-siRNA. In SSc-MSCs treated with scr-siRNA, the transforming growth factor (TGF)-β stimulus induces a significant decrease of CD248 expression. On the contrary, in CD248-siRNA cells, TGF-β is unable to modulate CD248 mRNA expression. **b** qRT-PCR of α-SMA in ten SSc-MSCs (five EOS and five LSS) transfected with specific CD248-siRNA or scr-siRNA. In SSc-MSCs treated with scr-siRNA, the TGF-β stimulus induces a significant increase of α-SMA mRNA expression. On the contrary, in CD248-siRNA cells, TGF-β is unable to modulate α-SMA mRNA expression. **c** qRT-PCR of Ki-67 in ten SSc-MSCs (five EOS and five LSS) transfected with specific CD248-siRNA or scr-siRNA. In SSc-MSCs treated with scr-siRNA, the PDGF-BB stimulus induces a significant increase of Ki-67 mRNA expression. On the contrary, in CD248-siRNA cells, PDGF-BB is unable to modulate Ki-67 mRNA expression. The TGF-β isoform used is TGF-β1. Any single dot in the figure represents the median of triplicate experiments for each patient. *** *p* = 0.0001. **d** Western blot analyses performed in four SSc-MSCs (two EOS and two LSS) confirmed the results observed by qRT-PCR analyses. Pictures are representative of all experiments. **e** and **f** Densitometric analysis of (**e**) CD248 protein bands and (**f**) α-SMA protein bands. The values were expressed as protein relative quantification/β-actin relative quantification. * *p* = 0.02

To address the role of CD248 in this cytokine network, we inactivated CD248 gene product in SSc-MSC by transfecting these cells with CD248-siRNA or scr-siRNA. CD248-siRNA efficiently knocked down CD248 molecules in SSc-MSC (> 71%), and, after silencing, TGF-β was unable to modulate the CD248 expression (Fig. 4a). Figure 4b shows that in CD248 silenced MSC, TGF-β stimulation did not induce α-SMA mRNA upregulation. Furthermore, in the same cells, PDGF-BB was unable to induce an increased expression of Ki-67 gene levels when compared with scr-siRNA-treated MSC (Fig. 4c). Western blot analysis confirmed the results of gene expression (Fig. 4d).

## Discussion

This report is the first, to the best of our knowledge, indicating that CD248, first identified as a tumor vascular endothelial antigen [35] and considered a key molecule of myofibroblast generation, is deeply involved in TGF-β and PDGF-BB signaling transduction during the fibrosis associated with SSc and that its inhibition strongly interferes with the profibrotic pathways of these two cytokines. Recently, CD248 has been considered as a marker of stromal fibroblasts, pericyte subsets, and human MSC [36, 37], and in the experimental model of fibrosis, after UUO, a significant upregulation of CD248 was observed [3]. On the contrary, CD248^−/−^ mice were protected from renal fibrosis and capillary rarefaction, probably inhibiting pericyte differentiation toward α-SMA^+^ interstitial myofibroblasts and preventing vascular instability and collagen production [3]. Furthermore, it has been reported that CD248 expressed on pericytes can promote EC apoptosis, probably impairing the cross-talk between EC integrins and vascular endothelial growth factor (VEGF) receptor 2, leading to the attenuation of VEGF signaling [38]. On these bases, CD248 may be considered a key target in those pathologic processes in which vascular damage and fibrosis are strongly joined in SSc.

In our present study, we chose to include in the patient cohort only patients affected by the Scl70^+^ diffuse form of the disease, because this subset rapidly progresses from vascular damage to fibrosis and may be considered a good “human model” to evaluate the link between vascular damage and fibrosis. In fact, it is well known that anti-Scl70 antibody is one of the typical autoimmune markers in SSc, occurring in 60.8% of cases of diffuse SSc and 23.4% of cases of limited SSc [39]. The presence of anti-Scl70 antibody is associated with severity of the disease, decreased survival [40], and evidence of pulmonary fibrosis [41]. We observed that CD248 was constitutively overexpressed on SSc cells derived from mesenchymal lineage. In SSc skin, we observed that CD248 was expressed on stromal fibroblasts and perivascular cells located in close proximity to the vessel, when compared with healthy skin, and the number of CD248^+^ cells significantly increased over time. Furthermore, in LSS-SSc biopsy, the CD248 mRNA expression of whole biopsy was significantly increased when compared with EOS-SSc. Of note, IF showed that the CD248^+^ cells coexpressed CD90, a molecule highly expressed in all the undifferentiated MSC [42, 43]. Currently, although we do not have a single cell marker capable of defining MSC, available literature suggests that CD90, which is highly expressed in all MSC, regardless of the source, may be considered a good marker to identify undifferentiated MSC [34]. The increased CD248 expression in perivascular cells of patients with SSc highly coexpressing the stem marker CD90 may suggest that in these MSC, both the profibrotic machinery and cells’ differentiation toward myofibroblasts may be activated.

Furthermore, our results showed that increased CD248 expression may be observed also in EC of SSc skin biopsies. It has been reported that CD248 may be expressed by endothelial progenitor cells [44] and tumor EC, together with pericytes and tumor-associated fibroblasts, during active cancer angiogenesis [38, 45]. It has been proposed that CD248 expressed by EC may interact with ECM proteins as well as tumor stromal cells to promote vascular invasion and migration [46]; in fact, CD248 expression was induced in EC when cultured in Matrigel, suggesting that endosialin could be induced in EC exposed to a complex extracellular environment [46]. In our setting, we hypothesize that SSc-EC may express CD248 as a compensatory mechanism to support angiogenesis in the context of a disease characterized by progressive desertification of vascular tree.

We investigated the functional role of CD248 in vitro in the perivascular differentiation toward myofibroblasts by using MSC, a validated surrogate of perivascular cells [26, 47]. Although the possible role of the CD248 molecule in SSc pathogenesis is largely unknown, it has been reported that this molecule is involved in the fibroproliferative process by modulating the PDGF-BB pathway [21] and collaborating with the TGF-β pathway to induce α-SMA expression [48]. In HC cells, TGF-β induced significantly decreased of CD248 when compared with UT cells. On the contrary, SSc-MSC displayed significantly higher CD248 expression than HC cells in both TGF-β-stimulated and unstimulated cultures. Recently, it has been shown that TGF-β strongly suppresses CD248 expression in healthy murine fibroblast cell lines; on the contrary, during cancer, where significantly higher CD248 levels are reported, mirroring what we observed in our cells, TGF-β failed to downregulate CD248 expression [49]. Although TGF-β is not a promoter of CD248 expression, CD248 may collaborate with TGF-β to induce α-SMA, possibly via downregulation of Notch3 [9, 12] and upregulation of interleukin 6 (IL6), C-C motif chemokine ligand 2 (CCL2), TGF-β1, and TGF-βR1 [48]. In fact, it has been reported that Notch3 may prevent the TGF-β1 induction of α-SMA, working as a molecular brake on smooth muscle gene transcription [50]. Furthermore, IL6, CCL2, TGF-β1, and TGF-βR1, activated by CD248, may strongly stimulate α-SMA [51]. Thus, we may hypothesize that CD248 overexpression in SSc-MSC may play its profibrotic role, exacerbating the TGF-β effects [52], by removing the Notch3 control and promoting α-SMA expression by its own downstream mediators. In fact, after silencing CD248, cells were unable to induce α-SMA expression after TGF-β stimulus, thus confirming the key role of CD248 in increasing the TGF-β effects in SSc-MSC.

To date, conflicting results have been reported in available literature concerning the expression of different smooth muscle genes during fibroblast proliferation and activation. Transcript levels of Sm22 are elevated in fibroblasts derived from mice lacking the cytoplasmic domain of CD248 [12], and CD248 knockdown did not affect the in vitro expression of α-SMA in normal human lung fibroblast [31]. On the contrary, in in vitro cultured human pericytes, the ability of TGF-β to induce α-SMA expression was lost in the CD248-specific siRNA-transfected primary human brain vascular pericytes, suggesting that α-SMA induction is CD248-dependent [53]. Furthermore, in an experimental model of liver fibrosis, the total liver mRNA of collagen and α-SMA was reduced in CD248-deficient mice compared with wild-type mice [19], and in a model of renal fibrosis, no increase of α-SMA molecule was observed in CD248-deficient mice [8]. These discrepancies may be partially explained by the different experimental models, the differences in cell manipulation, the intensity and quality of stimuli, and the different levels of efficacy in CD248 silencing.

To assess the contribution of CD248 in modulating the proliferative ability of SSc-MSC, we evaluated the expression of Ki-67 gene expression, which strongly correlates with cellular proliferation [19]. PDGF-BB induced a significant increase of Ki-67 levels when compared with UT cells in both HC- and SSc-MSC.

The role of CD248 in PDGF-BB signaling has been studied in human pericytes and hepatic stellate cells (HSC), commonly considered the precursors of septal myofibroblasts in liver fibrosis. PDGF-BB currently is considered one of the most potent HSC mitogens known, and it plays a key regulatory role in HSC proliferation during hepatic fibrogenesis [19]. It has been proposed that CD248 regulates the PDGF-BB pathway, thus controlling cell proliferation [54].

After CD248 silencing, we observed a strong inhibition of PDGF-BB-mediated cell proliferation, suggesting that this process is under CD248 control, thus decreasing the myofibroblast accumulation and confirming previous reports [23] in which primary renal fibroblasts isolated from CD248-knockout mice displays both decreased cell proliferation and reduced collagen secretion when compared with wild-type mice. Currently, it is still unclear how CD248 may modulate this proliferative signal: via the matrix-binding properties of CD248 and/or by the well-known ability of this molecule to potentiate PDGF-BB signaling. Recent studies in hepatic fibrosis, however, have provided evidence that HSC CD248^−/−^ display normal levels of PDGF receptors, suggesting that the antiproliferative effect of CD248^−/−^ HSCs is not mediated through the modulation of PDGF receptor expression [19].

## Conclusions

Our study shows that SSc perivascular cells overexpress CD248, which is involved in SSc pericyte transition toward myofibroblasts, and CD248 silencing may prevent pericyte-to-myofibroblast transition, proliferation, vascular instability, and tissue fibrosis. Taken together, our data suggest that targeting CD248 expression may be considered a potential target in order to block tissue fibrosis and vascular desertification during SSc. 

## Abbreviations

CCL2: C-C motif chemokine ligand 2
cDNA: Complementary DNA
ECM: Extracellular matrix
EC: Endothelial cell(s)
EOS: Early-onset subset
HC: Healthy control subject(s)
HSC: Hepatic stellate cell(s)
IF: Immunofluorescence
IL6: Interleukin 6
LSS: Long-standing subset
mRNA: Messenger RNA
MSC: Mesenchymal stem cell(s)
PDGF-BB: Platelet-derived growth factor BB
RP: Raynaud’s phenomenon
scr-siRNA: Nontargeting scrambled small interfering RNA
siRNA: Small interfering RNA
α-SMA: α-Smooth muscle actin
SSc: Systemic sclerosis
TGF-β: Transforming growth factor-β
UUO: Unilateral ureteral obstruction
VEGF: Vascular endothelial growth factor
vWF: Von Willebrand factor
